# Long-term outcomes of endoscopic submucosal dissection for superficial esophageal squamous cell carcinoma in Taiwan

**DOI:** 10.1186/s12876-021-01888-1

**Published:** 2021-08-03

**Authors:** Ming-Hung Hsu, Wen-Lun Wang, Tzu-Haw Chen, Chi-Ming Tai, Hsiu-Po Wang, Ching-Tai Lee

**Affiliations:** 1 Department of Internal Medicine, E-Da Hospital/I-Shou University, No. 1, Yida Road, Jiaosu Village, Yanchao District, Kaohsiung City, 82445 Taiwan; 2 Department of Internal Medicine, National Taiwan University Hospital, Taipei, Taiwan; 3 School of Medicine, College of Medicine, I-Shou University, Kaohsiung, Taiwan

**Keywords:** Esophageal squamous cell carcinoma, Endoscopic submucosal dissection, Lugol-voiding lesions

## Abstract

**Background:**

Endoscopic submucosal dissection (ESD) is gradually turning into the standard treatment for superficial esophageal squamous cell carcinoma (SESCC), however, the long-term outcomes have hardly ever been reported outside Japan.

**Method:**

We consecutively recruited patients with SESCC who had received ESD treatment at E-Da Hospital. The demographics, pathological characteristics, and Lugol staining background pattern (type A or B: none or < 10 small Lugol-voiding lesions [LVLs]; type C or D: > 10 small or multiform LVLs) were collected, and then correlated to outcomes and survival.

**Results:**

Total of 229 lesions were enrolled and the mean lesion size was 3.28 ± 1.69 (range 1–10) cm. 72% of the lesions had a type C-D Lugol staining background pattern. After ESD, the en bloc and R0 resection rates were 93.9% and 83.5%, respectively. Forty-nine subjects developed complications, including six (2.6%) with major bleeding, two (0.9%) with perforation, and 41 (17.9%) with strictures. Pathological staging showed that 19 cases had deep submucosal cancer invasion and subsequently received adjuvant therapies. During a mean follow-up period of 52.6 (range 3–146) months, 41 patients developed metachronous recurrence. The patients with a type C-D Lugol staining background pattern were associated with a higher risk of recurrence than those with few LVLs (log-rank *P* = 0.019). The 10-year survival rate was more than 90%, and only eight patients died of ESCC.

**Conclusion:**

ESD has excellent long-term outcomes but a high risk of metachronous recurrence. The Lugol staining pattern over the background mucosa could offer the risk stratification of metachronous recurrence.

## Introduction

Esophageal cancer has high mortality rate and also an increased incidence in some areas, especially Asia and Eastern Africa. The early detection and characterization of esophageal squamous cell carcinoma (ESCC) have ameliorated in recent years due to improvements in endoscopic technology such as image-enhanced endoscopy and magnifying endoscopy [1–5]. Endoscopic resection (ER), including endoscopic mucosal resection (EMR) and endoscopic submucosal dissection (ESD), has minimally invasive and potentially curative advantages for patients with superficial ESCC (SESCC) with a negligible risk of lymph node metastasis [6–10]. Compared to conventional EMR, ESD is generally acknowledged as a promising endoscopic treatment for SESCC, regardless of tumor size, with the advantages of higher en bloc and complete resection rates [11–13]. We recently reported a meta-analysis comparing the long-term outcomes of ESD and esophagectomy in treating SESCC, and found that ESD had similar efficacy to esophagectomy with far fewer lower perioperative adverse events. The 5-year overall, disease-specific and recurrence-free survival rates of ESD were 87.3%, 97.7% and 85.1%, respectively [14]. However, few studies except for Japan have reported the long-term outcomes of SESCC after ESD. In addition, even though the recurrence-free survival rate is suboptimal, the predictive factors for recurrence have yet to be unraveled. Therefore, the aims of this study were to assess the long-term outcomes of ESD for the treatment of SESCC in the largest reported cohort in Taiwan, and also to determine the risk factors for metachronous recurrence.

## Materials and methods

### Patients

This retrospective cohort study was conducted at a medical referral center in Taiwan. Patients with SESCC (clinical stage T1aN0) who were treated with ESD between June 2008 and August 2018 at E-DA Hospital were consecutively enrolled. All patients received optimal staging before treatment, including narrow band imaging, magnified endoscopy, Lugol chromoendoscopy (1.5% Lugol’s solution), endoscopic ultrasound, computed tomography (CT) and positron emission tomography (PET). All patients met the following eligibility criteria: histologically proven squamous cell carcinoma, tumor invasion depth limited in the mucosal layer, no lymph node or distant metastases and no prior chemo- or radiation therapy. A complete medical history was recorded, which included demographic and clinical data. Alcohol drinkers, betel nut chewers, and cigarette smokers were defined as those consuming any alcoholic beverage during the week, those who had chewed more than seven betel nuts per week, and those who smoked more than 10 cigarettes per week for at least 6 months, respectively [15]. The pattern of Lugol chromoendoscopy over the esophageal background mucosa was classified into four categories: type A or B: none or < 10 small Lugol-voiding lesions [LVLs]; type C or D: > 10 small or multiform LVLs, based on the criteria proposed by Muto and colleagues [16]. The Institutional Review Board of E-Da Hospital approved this study and all enrolled patients provided written informed consent before participating.

### ESD procedure

The ESD procedure was performed under general anesthesia as previously described [17].

First, Lugol was applied through a spray to delineate the margin of the lesion. Argon plasma coagulation (ERBE, Tuebingen, Germany) was then used to mark spots at 2–3 mm outside the margin of the esophageal neoplasia to guarantee a cancer-free margin. A 23-gauge disposable syringe was then used to inject 3–5 ml glycerol solution plus indigo-carmine with 0.0025% epinephrine into the submucosa to lift the lesion. A circumferential incision was made first, followed by submucosal dissection with an IT-knife 2 (KD-610L; Olympus Co. Ltd., Tokyo, Japan). Hemostatic forceps (FD-410LR; Olympus) were used to control bleeding in a soft coagulation mode (60-W output). During the whole procedure, carbon dioxide was used as insufflating gas.

An en bloc resection was defined as a one-piece resection including all marks. An en bloc resection with histologically cancer-free margins was defined as complete resection (R0) [18]. Immediate complications including massive bleeding, emphysema, and perforation during the ESD procedure were recorded. Latent complications including delayed bleeding and perforation after ESD were also recorded. Massive bleeding was defined as blood loss more than 500 ml or a hemoglobin drop of more than 2 g/dl. Perforation was diagnosed when other organs, extra-luminal fat, or a space was observed endoscopically through a visual hole in the esophageal wall. Mediastinal emphysema was defined as the presence of air in the mediastinal space. If mediastinal emphysema was suspected by clinical symptoms, CT of the chest would be arranged. Esophageal stricture was defined as narrowing with dysphagia and confirmed by conventional endoscopy with 9.8 mm outer diameter which cannot pass through, that required endoscopic treatment.

### Histologic assessment after ESD

The resected specimens were stretched and fixed on a piece of board and then bathed in 4% formalin for the final pathologic examination. After being embedded in 100% paraffin, all specimens were serially sectioned perpendicularly at 2-mm intervals and stained with hematoxylin and eosin. The histopathological evaluation of the resected specimen involved tumor size, depth of invasion, lymphovascular invasion (LVI), grade of differentiation, and resection margins. The depth of invasion was classified into five categories as intraepithelial cancer (m1), lamina propria (m2), muscularis mucosae (m3), and invading the superficial submucosa (≤ 200 μm, sm1) and deep submucosa (> sm1).

### Follow-up of the patients

If there was an incomplete resection margin histologically or poor pathological features (LVI, or deep submucosal invasion), adjuvant therapy (surgery or chemoradiation) was suggested. The patients in whom ESD was considered to be curative treatment underwent upper gastrointestinal endoscopy and regular clinical visits every 3 months during the first year. After the first year, the patients were examined every 6 months and then annually. Lugol chromoendoscopy was conducted during follow-up, and endoscopic biopsies were taken from any Lugol-voiding areas to identify any residual or recurrent neoplasia. Besides, CT of the chest and abdomen was also arranged to detect metastasis annually. Local recurrence was defined as a tumor which developed over the initial treatment area or besides a previous ESD scar. Metachronous recurrence was defined as a tumor which recurred at a new site after more than 6 months of complete remission status. Repeated endoscopic treatment, chemoradiotherapy or surgery would be arranged according to the tumor, node, metastasis (TNM) stage. Post-ESD survival was evaluated through regular clinic visits, medical records, or telephone contact until December 2019.

### Statistical analysis

All statistical analyses were performed using SPSS software (SPSS for Windows, version 18.0; SPSS Inc., Chicago, IL, USA). Cumulative cancer-related survival rates were estimated using Kaplan–Meier curves and assessed using log-rank tests. Logistic regression and Cox proportional hazard analysis were used to assess the factors associated with metachronous cancer development and a worse outcome, respectively. A *p-*value less than 0.05 was considered to indicate a statistically significant difference.

## Results

### Demographics and endoscopic characteristics

A total of 229 early-stage esophageal neoplasias in 167 patients (160 males; 7 females) were enrolled. The demographic characteristics of these patients are presented in Table 1. The mean age of the patients was 54 (range, 30–87) years. Smoking (89.8%), alcohol drinking (89.8%), and betel nut chewing (65.9%) were prevalent social behaviors. A total of 103 (61.7%) patients had a history of head and neck cancers. The endoscopic and pathological characteristics of these lesions are summarized in Table 2. The mean tumor size was 32.8 ± 16.9 mm (range, 10–100 mm). Overall, six (2.6%) tumors were located in the cervical esophagus, 36 (15.7%) in the upper esophagus, 127 (55.5%) in the middle third of the esophagus, and 60 (26.2%) in the lower-third of the esophagus. Ninety-three (45.0%) extended to more than one-half of the circumference, and 72% of the lesions demonstrated a type C or D Lugol staining background pattern.

**Table 1:** Clinical features of 229 lesions in 167 patients

Variable	Patient No. (%)
**Age**, years, mean ± SD	54 (30–87)
**Sex**	
Male	160 (95.8%)
Female	7 (4.2%)
**Smoking**	150 (89.8%)
**Alcohol drinker**	150 (89.8%)
**Betel nut chewing**	110 (65.9%)
**History of head and neck cancer**	103 (61.7%)
Oral cancer	30 (18.0%)
Oropharyngeal cancer	32 (19.2%)
Hypopharyngeal cancer	58 (34.7%)
Laryngeal cancer	7 (4.0%)

**Table 2:** Endoscopic and pathological characteristics

Variable	Lesion No. (%)
**Tumor size**, mm, mean ± SD	32.8 ± 16.9 (range:10–100)
**Tumor location**	
Cervical esophagus	6 (2.6%)
Upper third of the esophagus	36 (15.7%)
Middle third of the esophagus	127 (55.5%)
Lower third of the esophagus	60 (26.2%)
**Circumferential extension of the tumor**	
< 1/2	126 (55.0%)
< 3/4	74 (32.3%)
≥ 3/4	29 (12.7%)
**Lugol staining background**	
Type A or B	65 (28.4%)
Type C or D	164 (71.6%)
**Final pathology**	
High-grade dysplasia	131 (57.2%)
Squamous cell carcinoma	98 (42.8%)
**Depth of tumor invasion**	
Mucosa	189 (82.6%)
m1	100 (43.7%)
m2	60 (26.2%)
m3	29 (12.7%)
Submucosa	40 (17.4%)

### Short-term outcomes and procedure-related complications

After ESD, en bloc resection of the lesion was achieved in 215 (93.9%) lesions, and R0 resection in 191 (83.5%) lesions. The short-term outcomes and procedure-related adverse events are shown in Table 3. Eight patients (3.5%) developed ESD-related complications, including two with massive bleeding during ESD, four with delayed bleeding, and two with perforation during the procedure. Two patients with major adverse events received further surgery, and the others resolved after supportive treatment. There were no cases of procedure-related mortality. Forty-one patients (17.9%) developed post-ESD esophageal strictures which needed repeated balloon dilatation to resolve the symptoms.

**Table 3:** Short-term outcomes and complications after ESD

Variable	Patient No. (%)	Lesion No. (%)
**Diameter of the resected specimen**, mm, mean ± SD		32.8 ± 16.9 (range 10–100)
**En-bloc resection**		215 (93.9%)
**R0 resection**		191 (83.5%)
**ESD-related complication**		
Massive bleeding during ESD		2 (0.9%)
Delayed bleeding		4 (1.7%)
Perforation during ESD		2 (0.9%)
**Esophageal stricture needing balloon dilatation**		41 (17.9%)

^a^Mean follow-up period of 49.1 months (range 6–145 months)

ESD, endoscopic submucosal dissection

Post-ESD histological assessments showed that 131 (57.2%) lesions were high-grade dysplasia and 98 (42.8%) were squamous cell carcinoma. Regarding the depth of invasion, intraepithelial cancer (m1) was diagnosed in 100 (43.7%) lesions, cancer invading the lamina propria mucosae (m2) in 60 (26.2%) lesions, cancer invading the muscularis mucosae (m3) in 29 (12.7%) lesions, and cancer invading the submucosa in 40 (17.4%) lesions. There were only three patients that did not receive adjuvant therapy due to rapid progressing head and neck cancer. The remaining patients received surgery or chemoradiation for adjuvant therapy.

### Long-term outcomes of ESD

**Fig. 1:**
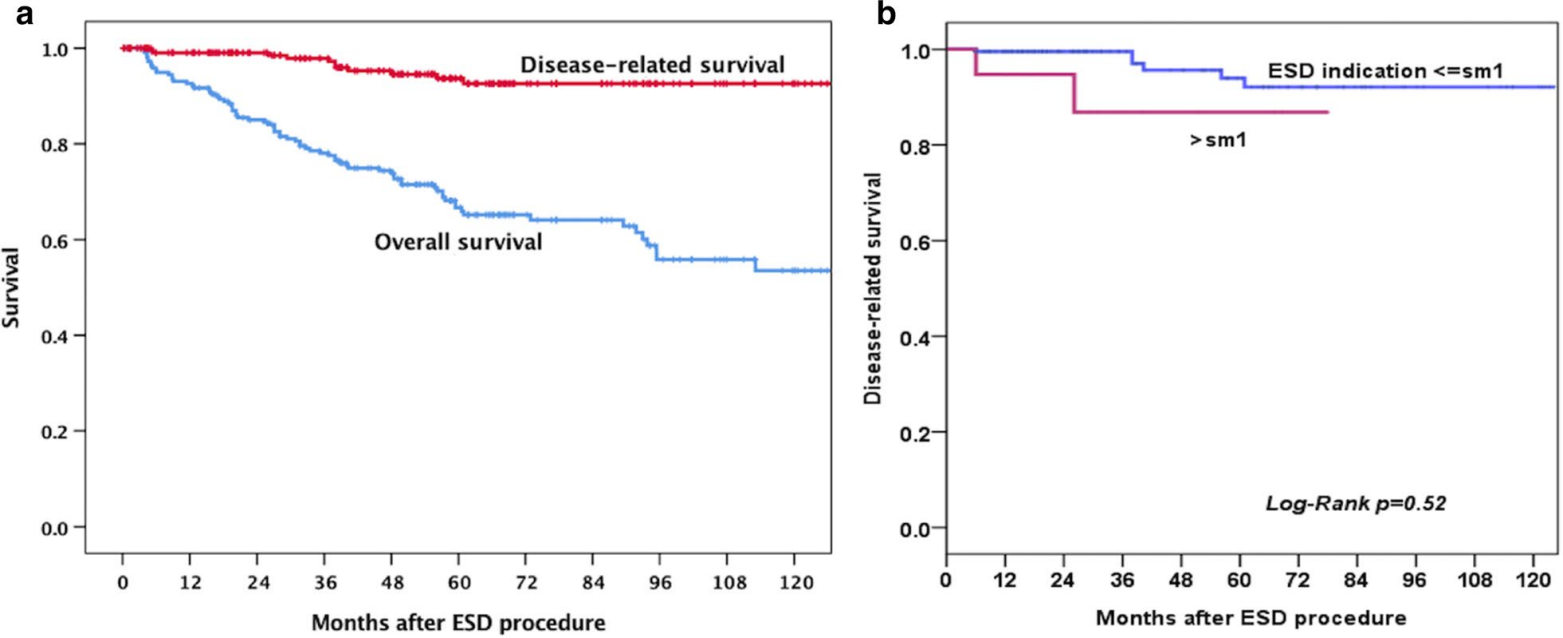
Kaplan–Meier 10-year survival curves for patients with SESCC (**A**) and patients with or without deep submucosal cancer invasion (> sm1) (**B**)

**Fig. 2:**
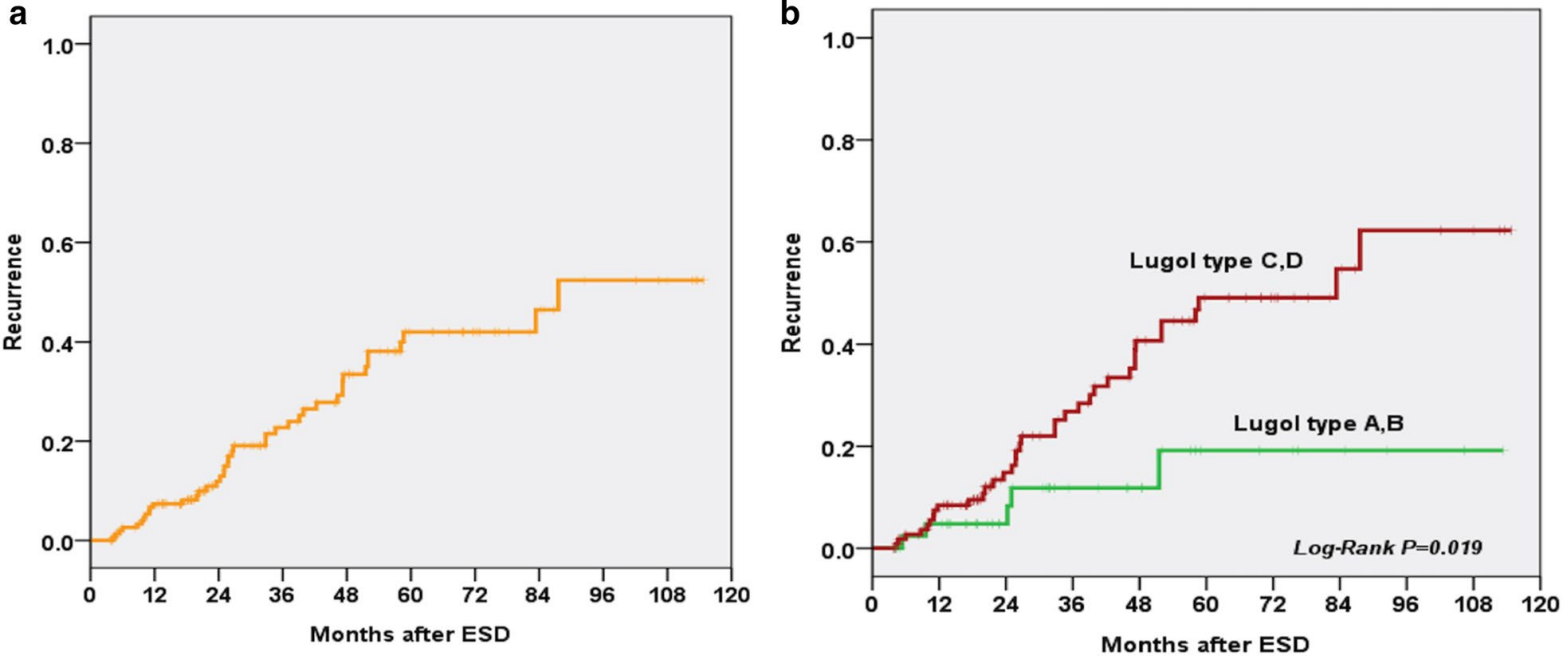
Recurrence curves of patients with SESCC (**A**) and subgroup analysis according to the Lugol staining pattern over the background mucosa (**B**)

During the follow-up period (mean 52.6 months; range, 3–146 months), 10 patients developed local recurrence (5.9%) who received repeated ESD and eight patients (4.5%) developed advanced cancer recurrence (4 suffered from regional lymphoid node metastasis, 2 developed distal metastasis, and 2 had both regional lymphoid node and distal metastasis) due to refusal of regular follow up. All of them received palliative chemotherapy and died of ESCC due to tumor progression. Besides, forty-one patients developed metachronous recurrence (17.9%), including 38/41 patients received endoscopic treatment (ESD, radiofrequency ablation or argon plasma coagulation) for mucosal or superficial submucosal recurrence. The remaining 3 patients received surgery or chemoradiation therapy due to advance stage. The ESCC-specific and overall survival is shown in Fig. 1. The 10-year disease-related survival rate of all patients was more than 90%, but the overall survival rate was lower than 70%. In subgroup analysis, the 10-year survival rate of the patients with deep submucosal cancer invasion (> sm1) who received adjuvant therapy was more than 85% (Fig. 1B). Notably, the metachronous recurrence rate was around 40% at 5 years in our ESD cohort (Fig. 2A).

**Fig. 3:**
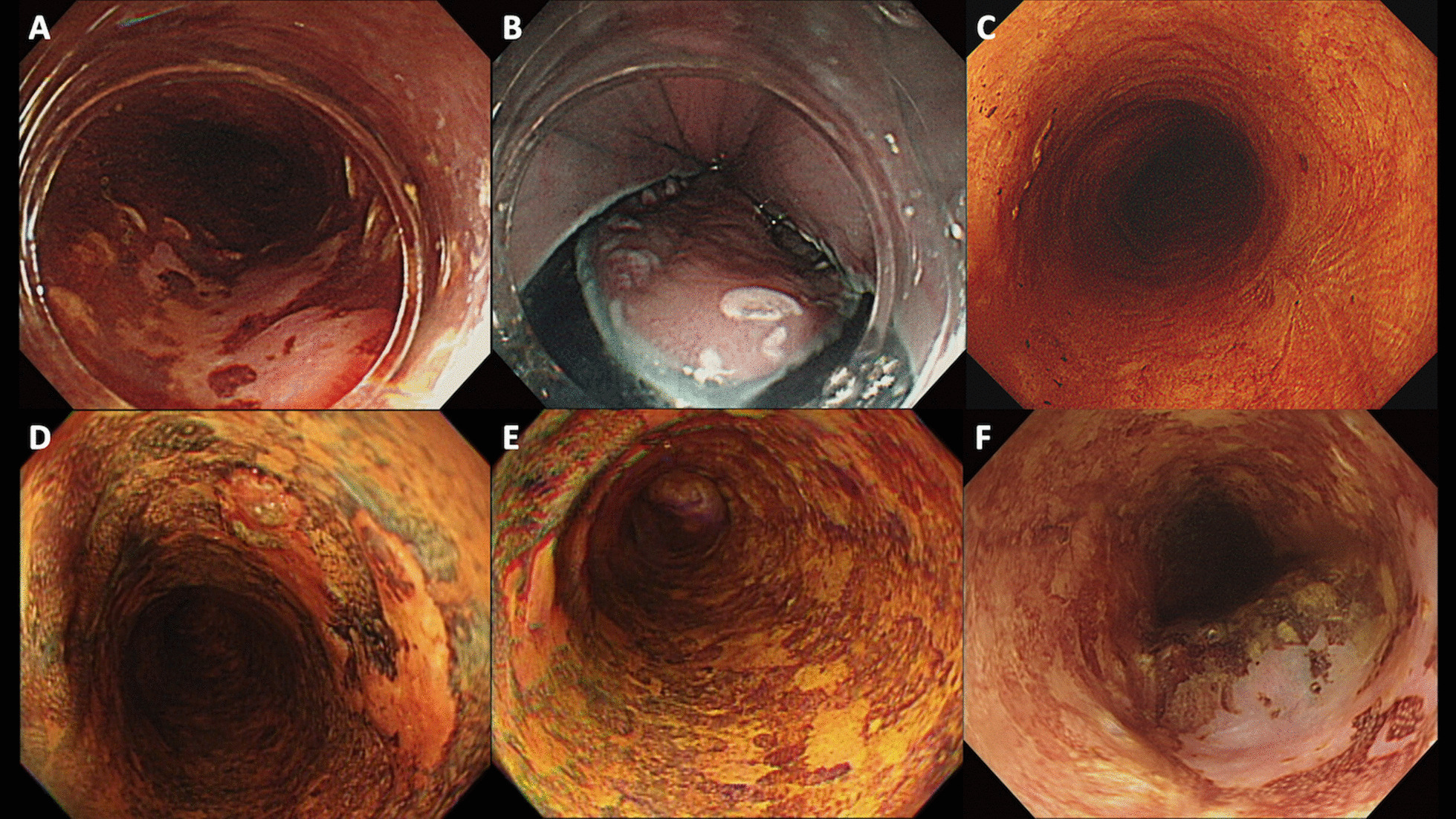
The series changes of two lesions with different Luogl staining background after ESD. **A** The main lesion with type A Lugol staining background. **B** Endoscopic appearance during endoscopic submucosal dissection. **C** Scar formation without recurrence. **D** The main lesion with type D Lugol staining background. **E** Type D Lugol staining background. **F** Metachronous recurrence after endoscopic submucosal dissection

Multivariate Cox regression analysis identified that the presence of numerous LVLs (type C or D pattern) was an independent predictive factor for metachronous recurrence (hazard ratio = 2.36; 95% confidence interval: 1.11–5.02, *P* = 0.025; Table 4**)**. The patients with numerous LVLs over the background mucosa (type C or D) were associated with a significantly higher metachronous recurrence rate than those with few LVLs (log-rank *P* = 0.019, Fig. 2B). The series changes of two lesions with different Luogl staining background after ESD are shown in Fig. 3.

**Table 4:** Cox regression model to assess the risk factors of metachronous recurrence

Factor	Variable	Case No	Hazard ratio	95% CI	*P* value
**Univariate analysis**					
Infiltration depth	< SM vs. > = SM	229	0.64	0.27–1.49	0.297
Size of tumor, cm	< 3 vs. > = 3	229	0.73	0.40–1.36	0.326
Lymphovascular involvement	+ vs. -	229	0.32	0.04–2.23	0.244
Age, years	< 50 vs. > = 50	229	0.56	0.33–0.94	**0.027**
Complete resection	+ vs. -	229	1.17	0.59–2.32	0.657
Lugol background	Few LVLs vs. numerous LVLs	229	2.56	1.21–5.41	**0.014**
**Multivariate analysis**					
Age, years	< 50 vs. > = 50	229	0.61	0.36–1.04	0.067
Lugol background	Few LVLs vs. numerous LVLs	229	2.36	1.11–5.02	**0.025**

## Discussion

Endoscopic submucosal dissection has gradually become the standard and potentially curative treatment for SESCC, even though it is a technically demanding procedure. In this study, we reported the short-term outcomes, complications and long-term outcomes of ESD for SESCC in the largest reported cohort in Taiwan. We showed that ESD is a safe and effective procedure, but that it is associated with a high metachronous recurrence rate (40% at 5 years). In addition, we found that Lugol staining before or during the ESD procedure to characterize the background mucosal pattern could help to stratify the risk of metachronous recurrence. Our findings not only validate the efficacy and safety of ESD in treating SESCC, but also provide evidence to guide surveillance after ESD.

We recently reported a meta-analysis to evaluate the efficacy and safety of ESD for SECC, in which the mean en bloc and R0 resection rates for ESD were 97.1% and 92.0%, respectively. The most commonly reported adverse events after ESD were perforation/mediastinal emphysema (3.4%), bleeding (2.0%), and stricture formation (9.4%) [14]. In the present study, the complete resection rate was more than 85% and the complication rate was less than 5%, indicating the efficacy and safety of the ESD technique at our high-volume referral center. Post-ESD stricture is still a major concern when treating lesions with a large circumferential extension, especially those occupying more than three-fourths of the circumference [19–22]. Even though we prescribed oral prednisolone (30 mg) for 4–8 weeks, 41 lesions developed post-ESD esophageal strictures which required endoscopic balloon dilatation in this study. Many methods have been developed to prevent the problem of post-ESD stricture, including pharmacological prophylaxis, mechanical strategies, tissue engineering strategies, and autologous transplantation. Among these methods, the use of steroids is currently the most commonly applied strategy to prevent esophageal post-ESD stricture, however these methods cannot completely prevent stricture in all patients [23, 24]. Sakaguchi et al. combined steroid injections and the shielding method with polyglycolic acid sheets which showed effectiveness in preventing post-ESD stricture. However, further studies are needed to evaluate whether polyglycolic acid (PGA) shielding has an additional effect on steroid injections [25], and a more effective and safe method to prevent stricture is still needed.

Metachronous recurrence cannot be totally avoided after ESD, and an increasing amount of research is focusing on this issue. In our cohort, 41 patients developed metachronous recurrence (17.9%), and the metachronous recurrence rate at 5 years was 40%, which is similar to previous reports [26, 27]. Furthermore, we identified that multiple small LVLs (> 10 in number) over the background mucosa was a predictor for metachronous recurrence. This method is useful and can be performed before or during the ESD procedure. In this study, we characterized the Lugol staining pattern into two types, few and multiple LVLs, which would be easier to use in clinical practice, compared to a previous study [28]. Future studies to treat the high-risk background are needed to prevent recurrence.

In this study, more than 70% of the patients had multiple LVLs in the background mucosa and 61.7% also had a history of head and neck cancers. The high metachronous recurrence rate in our cohort may be partly explained by a field cancerization phenomenon [29]. Waridel et al. first reported that inactivation of the p53 tumor suppressor gene and proliferation of cells containing p53 mutations are important biological steps in field cancerization [30]. Kobayashi et al. later also proved that p53 point mutations not only exist in high-grade dysplasia/carcinoma in situ, but also in a large number of low-grade dysplasias [31]. Muto et al. also reported that the aldehyde dehydrogenase 2 (ALDH2)-deficient genotype was associated with the appearance of numerous background LVLs [32, 33]. Subsequently, a meta-analysis also confirmed that polymorphisms of the ALDH2 gene were related to ESCC [34].

Besides, the present 10-year disease-related survival rate of all patients was more than 90% which was similar to those found in the previous studies from Japan [26, 35–37]. But, the overall survival rate was lower than 70%. The main difference between our study and the reports from Japan is that our patients had much higher (approximately 60%) concomitant head and neck cancer. The incidence of head and neck cancers is high in Taiwan due to non-negligible population with behavior of alcohol drinking, betel nut chewing, cigarette usage. As previous studies reported, upper aerodigestive tract squamous neoplasm shared the common risks. In our hospital, regular screening of esophageal squamous neoplasm was suggested for patients with head and neck cancers for decades. Survival of patients with esophageal squamous cell neoplasm (ESCN) significantly decreased when patients with second primary cancer (such as lung cancer, head and neck cancer, etc.) as Sato et al. reported [38]. Other studies also demonstrated that the location of head and neck cancer and type C or D Lugol staining background were also risks of synchronous ESCN [15, 16, 39, 40, 41]. As a result, although the present 10-year disease-related survival rate was similar to the reports from Japan, the overall survival rate was lower than 70% when consideration of the impact of concomitant head and neck cancer. In our study, more than 60% of patients had a history of head and neck cancers and the tumor location was more than 50% at the oropharyngeal and hypopharyngeal areas.

The current study has several limitations. First, this was a single-center, retrospective study. Second, the sample size was not large, and this may have affected the parameters of long-term outcomes. Third, the clinical outcomes of ESD were based on the experience of the endoscopists, which may have caused a limitation to external validity. Although our study provides data showing favorable long-term outcomes, a multicenter, prospective study is required.

In conclusion, ESD is a feasible and effective treatment option for superficial esophageal neoplasia. Although esophageal stricture is a major concern after ESD, it can be managed by endoscopic balloon dilatation. Hence, ESD should be suggested as a preferred treatment option for lesions amenable to endoscopic treatment. In addition, multiple LVLs over the background mucosa was an important predictive factor for metachronous recurrence, and this can be used to guide surveillance programs after ESD.

## Data Availability

The data-sets used and/or analysed during the current study available from the corresponding author on reasonable request.

## Abbreviations

ESCC: Esophageal squamous cell carcinoma
ER: Endoscopic resection
EMR: Endoscopic mucosal resection
ESD: Endoscopic submucosal dissection
SESCC: Superficial esophageal squamous cell carcinoma
CT: Computed tomography
PET: Positron emission tomography
LVLs: Lugol-voiding lesions
PGA: Polyglycolic acid
ALDH2: Aldehyde dehydrogenase 2
TNM: Tumor, node, metastasis
ESCN: Esophageal squamous cell neoplasm
